# In vivo and in silico studies on the potential role of garden cress oil in attenuating methotrexate-induced inflammation and apoptosis in liver

**DOI:** 10.1038/s41598-025-89550-8

**Published:** 2025-02-20

**Authors:** Dalia M. Mabrouk, Radwa H. El-Akad, Ahmed H. Afifi, Hafiza A. Sharaf, Sonia L. El-Sharkawy, Aida I. El makawy

**Affiliations:** 1 Cell Biology Department, Biotechnology Research Institute, National Research Centre, P.O.12622, Giza, Egypt; 2 Pharmacognosy Department, Pharmaceutical and Drug Industries Research Institute, National Research Centre, PO Box 12622, Cairo, Egypt; 3 Pathology Department, Medical Research and Clinical Studies Institute, National Research Centre, P.O.12622, Giza, Egypt

**Keywords:** Methotrexate, Hepatotoxicity, Garden cress, In silico, Inflammation, Apoptosis, Biochemistry, Cell biology, Computational biology and bioinformatics, Genetics

## Abstract

Methotrexate (MTX) has been used in high doses for cancer therapy and low doses for autoimmune diseases. It is proven that methotrexate-induced hepatotoxicity occurs even at relatively low doses. It is known that garden cress has anti-inflammatory, antioxidant, and hepatoprotective properties. This study investigates the potential alleviating effect of garden cress oil (GCO) against MTX-induced hepatotoxicity in rats. The chemical composition of GCO was assessed using GC/MS analysis. Liver damage was studied using hepatotoxicity biomarkers, molecular, and histological analysis. Also, the effects of GCO on TNF-α and caspase-3 proteins were evaluated through molecular docking studies. The results demonstrated that MTX caused liver damage, as seen by elevated levels of the liver enzymes ALT, AST, and ALP. Likewise, MTX showed clear signs of apoptosis, such as increased mRNA expression levels of BAX, Caspase-3, and P53, and increased liver inflammation indicated by higher levels of TNF-α expression. MTX exhibited significant liver damage, as demonstrated by histological examination. Treatment with GCO effectively alleviated the apoptotic effects of MTX, provided protection against inflammation, and restored histological alterations. GC/MS metabolite profiling of garden cress oil revealed the presence of several phytoconstituents, including tocopherols, erucic acid, sesamolin, linoleic acid, vaccenic acid, oleic acid, stearic acid, and palmitic acid, that showed strong binding affinities toward TNF-α and caspase-3 proteins in molecular docking studies, which could explain the anti-apoptotic and anti-inflammatory potential of GCO.

## Introduction

Drug-induced liver injury is a significant issue that limits the duration of medication therapy and undermines its positive effects. These effects could potentially result from modifications in distinct pathways that trigger an immediate toxic effect, the production of active metabolites, or an immune response. Consequently, over the last decade, researchers, government agencies, medical professionals, and pharmaceutical companies have all paid close attention to the rise in drug-induced liver damage. Over a thousand medications have been linked to liver injury, which can lead to inflammation, liver cell death, and severe liver failure^1^.

Methotrexate (MTX) is one of the most effective and widely used drugs in the management of autoimmune diseases^2^. Methotrexate is specified for a variety of medical conditions, including autoimmune rheumatic, psoriatic and juvenile idiopathic arthritis, inflammatory myopathies, sarcoidosis, rheumatic polymyalgia, arthritis related to secondary amyloidosis, and others. It is also used for other autoimmune conditions, such as Sjogren syndrome, inflammatory bowel disease, vasculitis, and some neoplasms^3,4^. It is an antifolic acid medication derived from aminopterin that can inhibit DNA synthesis and repair^5,6^. One potential mechanism for MTX-induced liver damage is the prolonged presence of MTX in cells due to hepatocytes storing and metabolizing it in its polyglutamated form^7,8^. Disruption of the intestinal barrier function may lead to methotrexate-induced hepatotoxicity, allowing bacteria to translocate to the liver and cause damage. Furthermore, MTX has been shown to increase intestinal permeability, which is linked to elevated hepatic enzymes, fibrosis, cirrhosis, and hepatic inflammation^9^. Several studies have indicated that hepatotoxicity can result from an imbalance in the regulation of oxidative stress, inflammation, endothelial damage, and apoptosis^2,10–14^. Additionally, MTX may be used in lower dosages as a disease-modifying anti-rheumatic drug for autoimmune diseases, but this use can still result in liver damage as a side effect ^15^, indicating that MTX-induced hepatotoxicity may not be dose-dependent^16^.

Apoptosis is essential for maintaining tissue homeostasis in the body and occurs under the control of different genes in multicellular and unicellular organisms. The pro-apoptotic Bcl-associated X (BAX) protein and anti-apoptotic B-cell lymphoma 2 (Bcl-2) protein play significant roles in forming mitochondrial apoptotic channels. Caspase protease enzymes, including caspase-3 and 7, are also involved in various apoptotic pathways. The TP53 gene is one of the most widely studied genes in human cells due to its multifaceted functions and complex dynamics. P53 can induce apoptosis in a genetically unstable cell by interacting with many pro-apoptotic and anti-apoptotic factors^17,18^. In addition, TNFα, mainly produced by activated macrophages during inflammation, has been implicated as an important pathogenic mediator in liver diseases^19^.

It has been reported that plant-based medicines are a source of biologically active ingredients and are important in global healthcare, including alternative, conventional, and preventative medicine^20^. *Lepidium sativum* (LS) or Garden cress, a member of the Brassicaceae family, can be topically used to relieve rheumatism, inflammation, and sore muscles ^21^. Additionally, it has hepatoprotective, anti-diarrheal, antioxidant, blood-purifying, hunger-stimulating, antispasmodic, and anticancer properties^22,23^. It also possesses anti-diabetic, laxative, cholesterol-lowering, fracture-healing, pain-relieving, procoagulant, and diuretic properties. According to Emhofer et al.^24^, it contains proteins, vitamins, carbohydrates, omega-3 fatty acids, iron, phytochemicals, and flavonoids. The liver-protective properties of garden cress seeds have come to light due to their ability to promote liver function and protect it from injury. Therefore, many studies are investigating the bioactive compounds in garden cress seeds and their impact on liver function^25^. In conventional medicine, garden cress seed treat inflammatory conditions like diabetes mellitus, arthritis, and hepatitis^26^. Abdulaziz et al.^27^ found that garden cress strongly inhibits inflammation-progressing enzymes (COX1 and 2). Sayed et al.^28^ indicated that *Lepidium sativum* (LS) can modulate the expression of inflammation markers such as IL-6, TNF-α, IL-4, and IL-10 in lipopolysaccharide-induced liver damage in mice.

The purpose of this study was to explore three main objectives: 1) to examine the potential impact of garden cress oil on reducing hepatotoxicity caused by low-dose methotrexate in rats, 2) to analyze the chemical composition of GCO, and 3) to predict the binding modes and affinities of the major metabolites against potential biological targets using molecular docking tools.

## Results

### Lipid content determination in GCO via GC/MS analysis

**Fig. 1:**
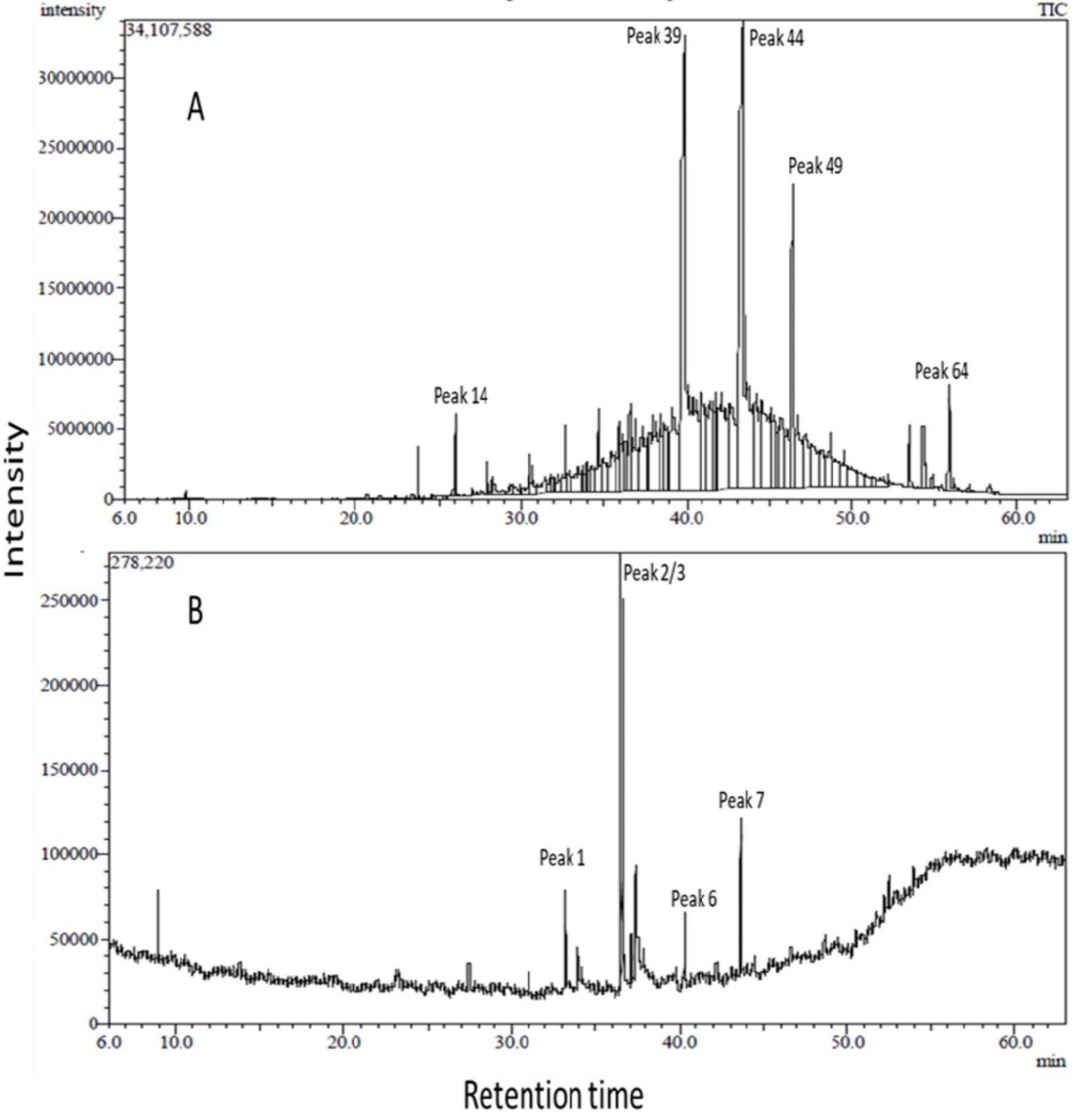
Total ion chromatograms of unsaponifiable matter (**A**) and fatty acid methyl esters (**B**) detected in garden cress seed oil (*Lepidium sativum*) via GC/MS analyses. Peaks follow the numbering of identified compounds in Table 1 and Table 2.

Total ion chromatograms of the analyzed unsaponifiable matter (unsap.) and FAME are shown in Fig. 1. The area percentage of identified compounds is presented in Table 1 and Table 2. Analysis of unsaponifiable matter revealed the identification of 67 metabolites constituting 89.5%; the majority of which are alkane hydrocarbons (80.26%) varying between branched and straight chain (Table 1). Herein, 36 alkane hydrocarbons were detected. Long chain unsaturated fatty alcohols docosenol (15.1%) and eicosenol (15.01%) were the major compounds in the analyzed sample (Table 1). Other detected compounds included alcohols, esters and aldehydes of saturated and unsaturated hydrocarbon chains varying from C-12 to C-31 (Table 1).

**Table 1:** Relative area percentage of unsaponifiable components detected in garden cress seed oil (*Lepidium sativum*) *via* GC/MS analysis.

Peak	Rt (min.)	RI_exp._	RI_theoretical_	Identification	Molecular weight	Molecular formula	Area %
Terpenes							
1.	6.8	955	940	α-Pinene	136	C_10_H_16_	0.016
2.	8.02	1040	1030	β-Phellandrene	136	C_10_H_16_	0.004
3.	9.15	1052	1038	Ocimene	136	C_10_H_16_	0.003
4.	9.6	1061	1042	Cymene	134	C_10_H_14_	0.009
5.	9.75	1063	1044	Limonene	136	C_10_H_16_	0.087
6.	9.82	1075	1059	Cineol	154	C_10_H_18_O	0.011
7.	21.45	1428	1433	Caryophyllene	204	C_15_H_24_	0.033
8.	21.8	1431	1442	Farnesene	204	C_15_H_24_	0.011
9.	23.08	1480	1500	Cuparene	202	C_15_H_22_	0.027
10.	23.2	1490	1505	Cuprenene	204	C_15_H_24_	0.011
11.	23.75	1500	1510	Bisabolene	204	C_15_H_24_	0.734
12.	24.14	1520	1522	Sesquiphellandrene	204	C_15_H_24_	0.027
13.	25.69	1579	1573	Caryophyllene oxide	220	C_15_H_24_O	0.066
14.	26	1607	1593	Carotol	222	C_15_H_26_O	1.1
15.	27.07	1651	1656	Daucol	238	C_15_H_26_O_2_	0.05
16.	36.92	2122	2118	Phytol	296	C_20_H_40_O	1.30
17.	48.63	2840	2790	Squalene	410	C_30_H_50_	1.04
18.	56.5	3401	3337	β-amyrin	426	C_30_H_50_O	0.04
19.	57.12	3440	3425	Cycloartenol	426	C_30_H_50_O	0.02
Total terpenes							4.75
Phenylpropanoids							
20.	20.3	1330	1319	Methyl cinnamaldehyde	146	C_10_H_10_O	0.06
21.	27.98	1691	1676	Asarone < (E)->	208	C_12_H_16_O_3_	0.44
Total phenylpropanoids							0.5
Alkane hydrocarbons							
22.	18.03	1110	1106	Dodecane	170	C_12_H_26_	0.002
23.	19.5	1255	1250	Methyl dodecane	184	C_13_H_28_	0.003
24.	20.8	1427	1413	n-Tetradecane	198	C_14_H_30_	0.02
25.	28.40	1709	1700	n-Heptadecane	240	C_17_H_36_	0.27
26.	29.6	1764	1746	2-Methylheptadecane	254	C_18_H_38_	0.2
27.	30.5	1801	1810	n-Octadecane	254	C_18_H_38_	0.36
28.	31.7	1861	1845	2-Methyloctadecane	268	C_19_H_40_	0.3
29.	32.63	1904	1910	n-Nonadecane	268	C_19_H_40_	0.75
30.	33.47	1945	1945	9-Methylnonadecane	282	C_20_H_42_	0.93
31.	34.85	2012	2009	n-Eicosane	282	C_20_H_42_	1.6
32.	35.8	2044	2045	10-Methylicosane	296	C_21_H_44_	1.07
33.	36.12	2079	2077	n-Octadecanol	270	C_18_H_38_O	1.4
34.	36.62	2106	2109	n-Heneicosane	296	C_21_H_44_	1.1
35.	37.64	2162	2173	3-Methylheneicosane	310	C_22_H_46_	3.4
36.	38.4	2204	2208	n-Docosane	310	C_22_H_46_	2.5
37.	38.8	2225	2210	Octadecanol acetate	312	C_20_H_40_O_2_	0.82
38.	39.1	2243	2246	6-methylDocosane	324	C_23_H_48_	2.87
39.	39.88	2285	2260	Eicosenol	296	C_20_H_40_O	15.01
40.	40.99	2349	2300	n-Tricosane	324	C_23_H_48_	2.62
41.	41.55	2379	2365	2-Methyltricosane	338	C_24_H_50_	2.37
42.	41.76	2393	2375	n-Heptadecylcyclohexane	322	C23H_46_	1.4
43.	42.11	2415	2400	n-Tetracosane	338	C_24_H_50_	3.68
44.	43.36	2492	2460	13-Docosenol	324	C_22_H_44_O	15.13
45.	44.18	2543	2500	n-Pentacosane	352	C_25_H_52_	2.14
46.	44.53	2565	2542	2-Methylpentacosane	366	C_26_H_54_	4.02
47.	45.29	2614	2600	n-Hexacosane	366	C_26_H_54_	1.99
48.	46.09	2667	2641	2-Methylhexacosane	380	C_27_H_56_	1.32
49.	46.40	2688	2650	Tetracosanal	352	C_24_H_48_O	3.51
50.	46.69	2741	2700	n-Heptacosane	380	C_27_H_56_	1.86
51.	47.3	2768	2740	2-Methylheptacosane	394	C_28_H_58_	3.8
52.	48.20	2809	2800	n-Octacosane	394	C_28_H_58_	1.32
53.	49.01	2868	2840	3-Methyloctacosane	408	C_29_H_60_	1.05
54.	49.45	2906	2900	n-Nonacosane	408	C_29_H_60_	0.71
55.	50.5	3004	3000	n-Triacontane	422	C_30_H_62_	0.36
56.	52	3080	3060	Hexamethyl-tetracosahexaen-3-ol	426	C_30_H_50_O	0.29
57.	52.25	3105	3100	n-Untriacontane	436	C_31_H_64_	0.09
Total alkane hydrocarbons							80.26
Tocopherols							
58.	51.6	3030	3040	β/γ-Tocopherol	416	C_28_H_48_O_2_	0.07
59.	52.91	3155	3138	α-Tocopherol (vitamin E)	430	C_29_H_50_O_2_	0.009
Total tocopherols							0.079
Sterols							
60.	52.83	3130	3110	Cholesterol	386	C_26_H_42_O_2_	0.01
61.	53.2	3165	3144	Ergosta-5,22-dien-3-ol	398	C_28_H_46_O	0.08
62.	54	3230	3165	Campesterol	400	C_28_H_48_O	0.188
63.	54.86	3292	3230	Stigmasterol	412	C_29_H_48_O	0.10
64.	55.4	3296	3235	Sitosterol	414	C_29_H_50_O	1.13
65.	56.09	3360	3315	Stigmasta-5,24(28)-dien-3-ol	412	C_29_H_48_O	0.09
Total sterols							1.59
Lignans							
66.	53.4	3199	3150	Sesamin	354	C_20_H_18_O_6_	0.97
67.	54.4	3256	3208	Sesamolin	370	C_20_H_18_O_7_	1.5
Total lignans							2.47
Total identified compounds							89.5

**Fig. 2:**
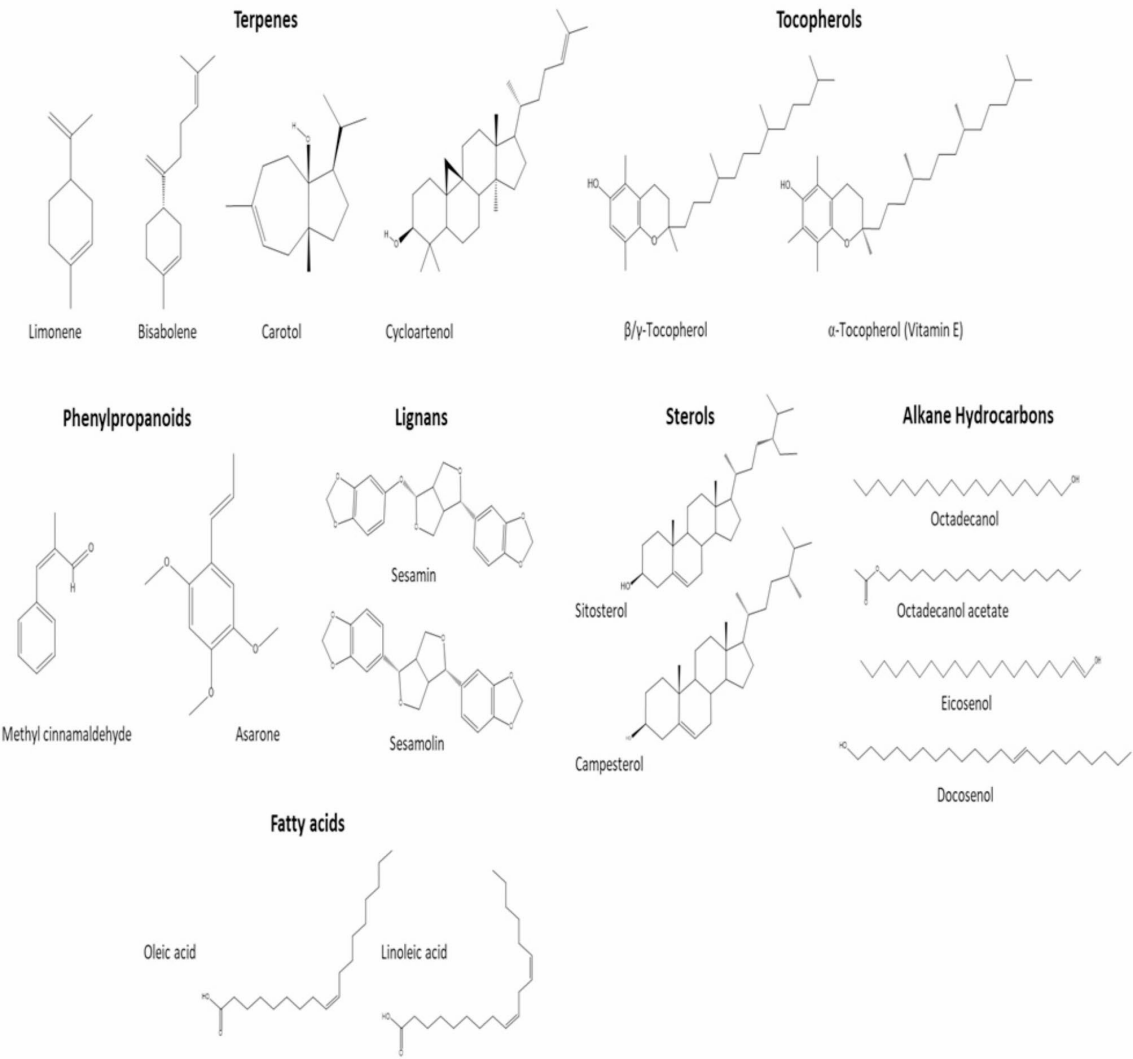
Structures of selected compounds identified in garden cress seed oil via GC/MS analysis of unsaponifiable matter and fatty acids (as methyl esters).

Other identified compounds belong to diverse phytochemical classes including 19 mono-/sesqui-/di-/and tri-terpenes (4.75%), 2 phenyl propanoids (0.5%), 3 tocopherols (0.08%), 2 lignans (Sesamin (0.97%) and sesamolin (1.5%) and 6 sterols (1.59%) (Fig. 2). Carotane sesquiterpenes, carotol (1.1%) and daucol (0.05%), are reported herein for the first time as well as bisabolene (0.73%), cuparene (0.027%) and cupranene (0.011%), Conversely, 6 fatty acids were identified as methyl esters (92.02%) that included oleic (33.1%) and linoleic acids (30.9%) followed by erucic, vaccenic, palmitic and stearic acids (Table 2).

**Table 2:** Relative area percentage of fatty acid methyl esters detected in garden cress seed oil (*Lepidium sativum*) via GC/MS analysis.

Peak	Rt (min.)	Identification	Area %
1.	33.1	Palmitic acid, methyl ester	6.85
2.	36.5	Linoleic acid, methyl ester	30.97
3.	36.7	Oleic acid, methyl ester	33.1
4.	37.1	Stearic acid, methyl ester	4.19
5.	37.38	Vaccenic acid, methyl ester	7
6.	43.6	Erucic acid, methyl ester	9.9
Total			92.02%
Saturated fatty acids			11.04%
Unsaturated fatty acids			80.98

### Molecular docking study

Molecular docking study was employed to assess the binding affinities and binding poses of the identified phytoconstituents in garden cress oil against the active sites of TNF-α (pdb: 7JRA) and Caspase-3 (pdb: 3GJQ) with the aim to predict the underlying mechanism of the hepatoprotective effect of garden cress oil against MTX-induced hepatotoxicity. Table 3 displays the binding energy of the top-scoring phytoconstituents against the two targets.

**Table 3:** Docking results scoring Garden cress oil identified phytoconstituents against TNF-α and Caspase-3 binding sites.

TNF-α (PDB: 7JRA)		Caspase-3 (PDB: 3GJQ)	
Ligand	Binding energy ΔG (kcal/mol)	Compound	Binding energy ΔG (kcal/mol)
Co-crystalized inhibitor	-12.89	Co-crystalized inhibitor	-11.01
β-Tocopherol	-10.73	Linoleic acid	-10.05
Erucic acid	-9.97	Vaccenic acid	-7.48
Sesamolin	-9.91	Oleic acid	-7.45
α-Tocopherol	-9.55	Stearic acid	-6.93
γ-Tocopherol	-9.52	Palmitic acid	-6.89

Validation of the docking protocol was assessed through redocking of the co-crystalized ligands against their corresponding proteins and calculating the RMSD value between the docked pose and the co-crystalized pose for both ligands. The validity of the docking parameters was donated by the excellent superposition and small RMSD value between docked and co-crystalized poses for both ligand (0.587 and 0.846 Å for TNF-α and Caspase-3, respectively).

**Fig. 3:**
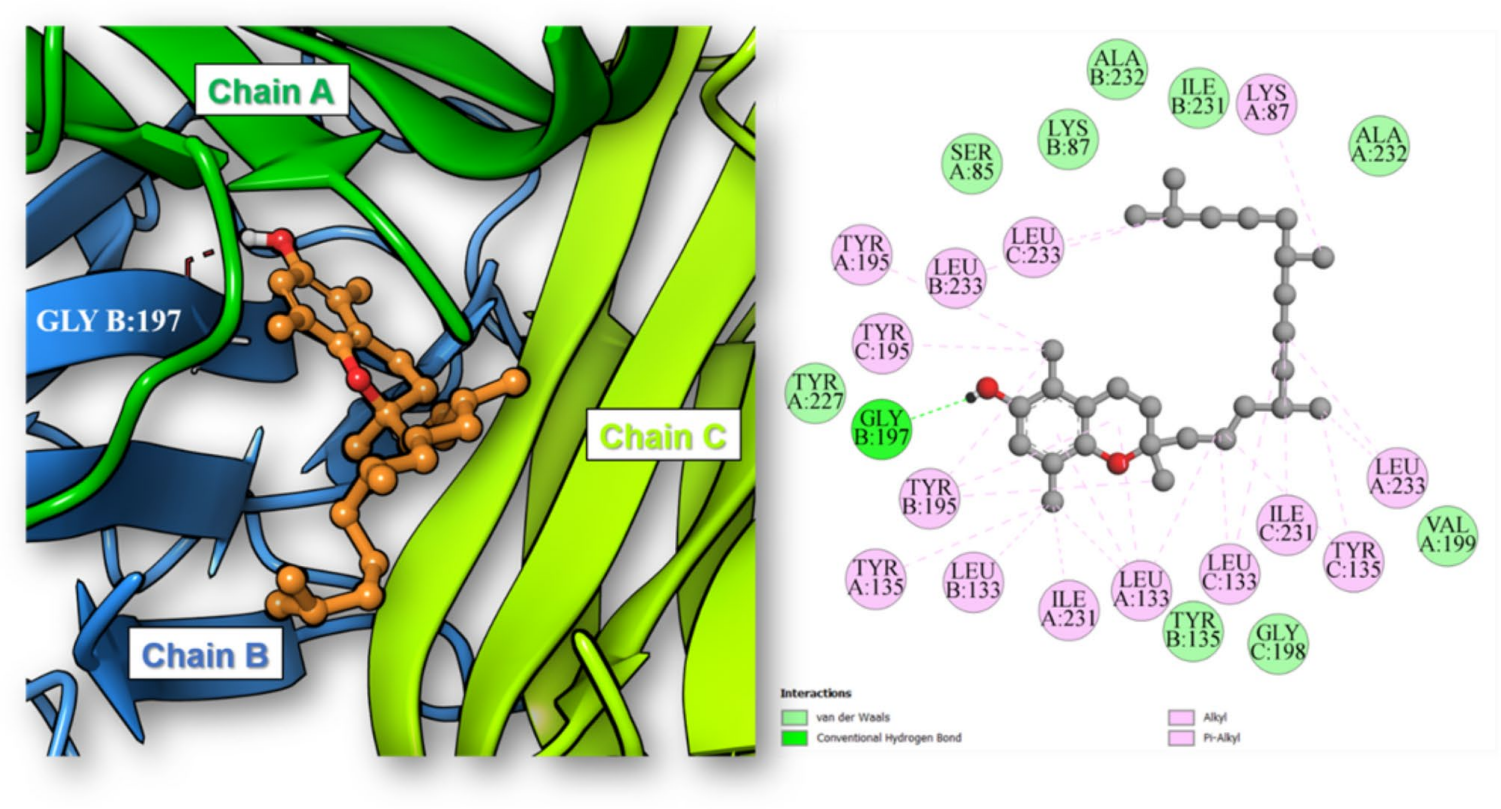
3D and 2D illustration of the docking pose and binding interactions of β-tocopherol (ball and sticks) in the active site of TNF-α homotrimer (pdb: 7JRA).

**Fig. 4:**
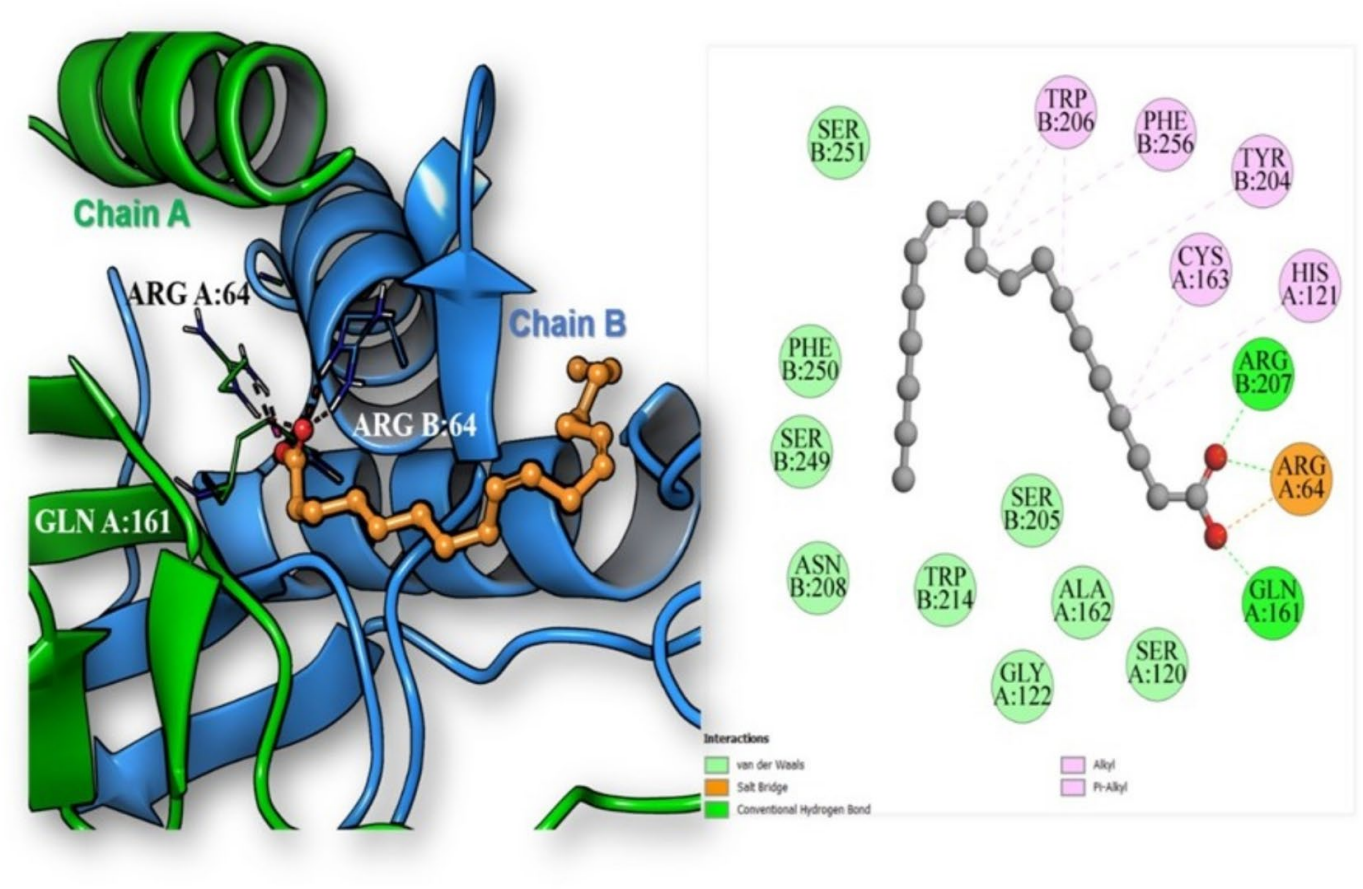
3D and 2D illustration of the docking pose and binding interactions of linoleic acid (ball and sticks) in the active site of caspase-3 heterodimer (pdb: 3GJQ).

Among the identified phytoconstituents, α, β and γ-tocopherols along with erucic acid and Sesamolin exhibited the highest binding affinities toward the TNF-α binding site with binding energies -9.55, -10.73, -9.52, -9.97 and − 9.91 kcal/mol, respectively. Inspection of the best scoring pose of β-tocopherol in the active site of the TNF-α homotrimer revealed the formation of one conventional hydrogen bond between the phenolic hydroxyl group of β-tocopherol and Gly197 residue in chain B of TNF-α. Moreover, several hydrophobic interactions have also contributed to anchor the compound in the hydrophobic binding cavity (Fig. 3). On the other hand, the highest binding affinities to the Caspase-3 active site were observed by linoleic acid, vaccenic acid, oleic acid, stearic acid and palmitic acid as they achieved binding energies of -10.05, -7.48, -7.45, -6.93 and − 6.89 kcal/mol, respectively. The best scoring pose of linoleic acid displayed its binding to the active site at the interface between p17 (chain A) and p12 (chain B) subunits of the caspase-3 heterodimer and the formation of three conventional hydrogen bonds by linoleic acid carboxylic group with the residues Arg64 (chain A), Gln161 (chain A) and Arg207 (chain B) in addition to one salt bridge with Arg64 (chain A). Also, several hydrophobic interactions with the active site residues were observed in (Fig. 4).

### Liver enzymes

**Fig. 5:**
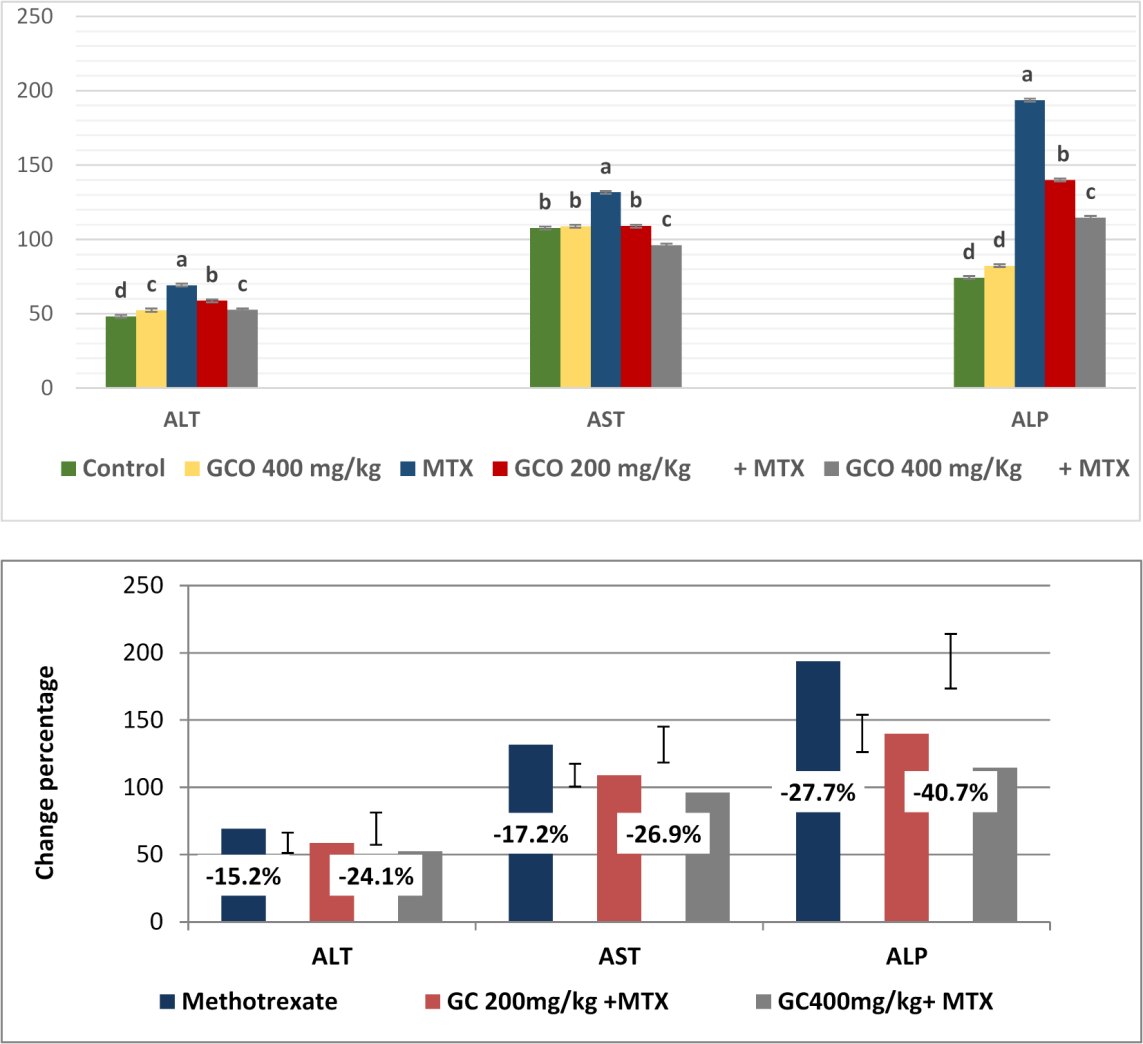
Effect of methotrexate and garden cress oil on liver enzymes. Data are expressed as mean ± SE (*n* = 6). Groups with unlike superscript letters in each raw were significantly different at (*P* ≤ 0.05).

The data analysis of the effect of methotrexate and garden cress oil on rat liver enzymes and the change percent between the MTX group and GCO two doses are shown in Fig. 5. Methotrexate significantly increased (*P* ≤ 0.05) serum ALT, AST, and ALP levels compared to the control group. In contrast, the administration of garden cress oil showed a significant dose-dependent decrease in ALT, AST, and ALP levels (*P* ≤ 0.05) compared to methotrexate.

### mRNA expression of inflammatory and apoptotic genes

**Fig. 6:**
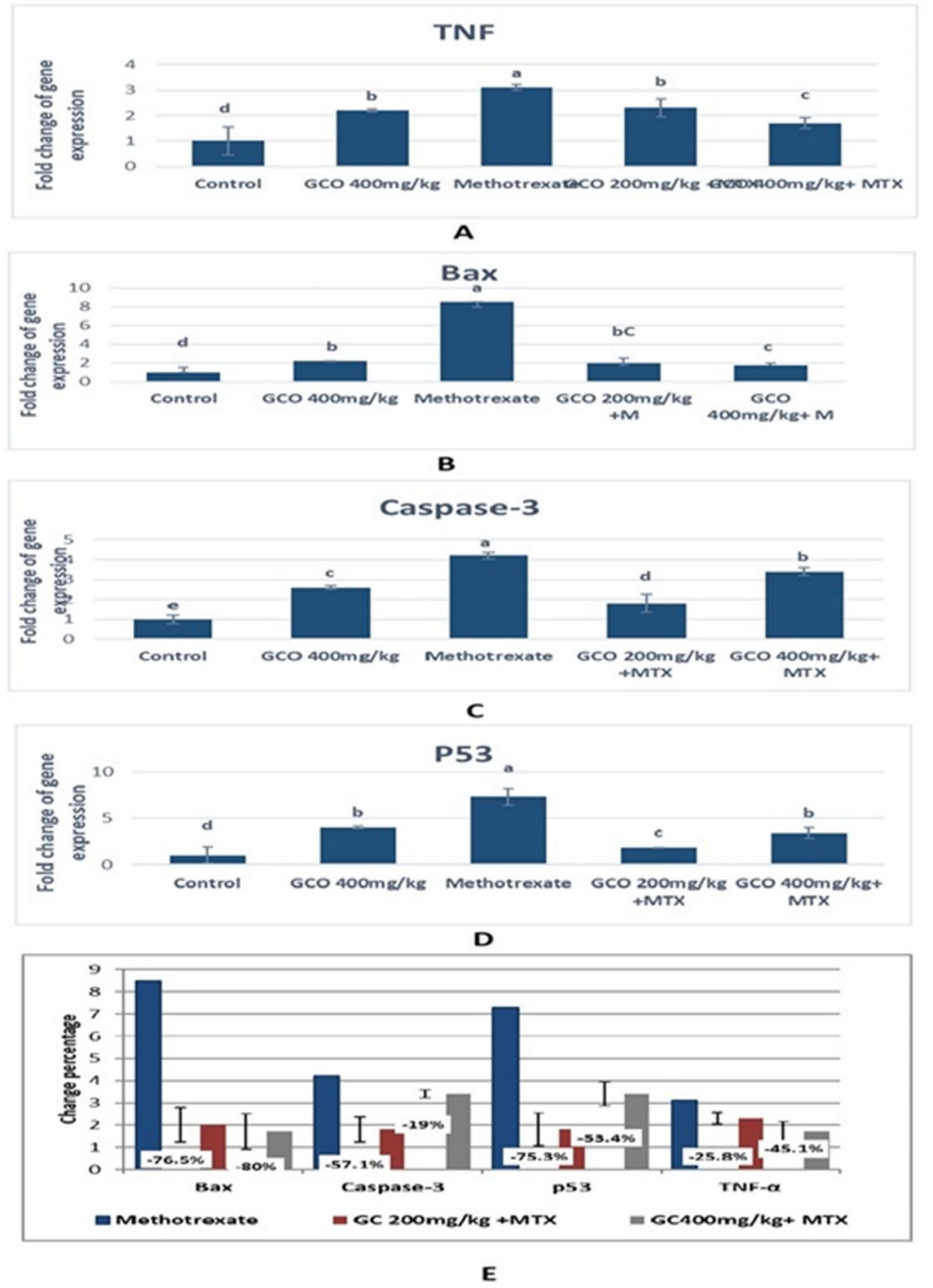
The relative fold changes of inflammatory and apoptotic genes expression and the percentage change between the methotrexate and the garden cress oil-treated groups. Data are expressed as mean ± SE (n = 3) for all tested groups. Groups with different superscript letters were significantly different (*P* ≤ 0.05).

Figures 6A–D present the Bax, Caspase-3, TNF, and TP53 gene expression in the liver of all experimental groups, and Fig. 6E denotes the comparison of changes in percentage gene expression between the methotrexate group and the garden cress oil-treated groups. Injection with MTX induced a significant increase in the mRNA expression level of TNFα, a proinflammatory marker, compared to the control groups (*P* ≤ 0.05). However, treatment with GCO (200 and 400 mg/kg) led to a significant downregulation (*P *≤ 0.05) of TNFα compared to the MTX-treated group. Nevertheless, TNFα expression remained significantly higher among rats treated with GCO/MTX compared to the control group (Fig. 6A). Next, we analyzed the expression levels of the pro-apoptotic genes (Bax, P53, Caspase-3). The results showed that MTX caused a significant increase in Bax, P53, and Caspase-3 expression (*P* ≤ 0.05). However, treatment with GCO (200 and 400 mg/kg) effectively reversed these effects (*P* ≤ 0.05) (Fig. 6B–D).

## Histological results

**Fig. 7:**
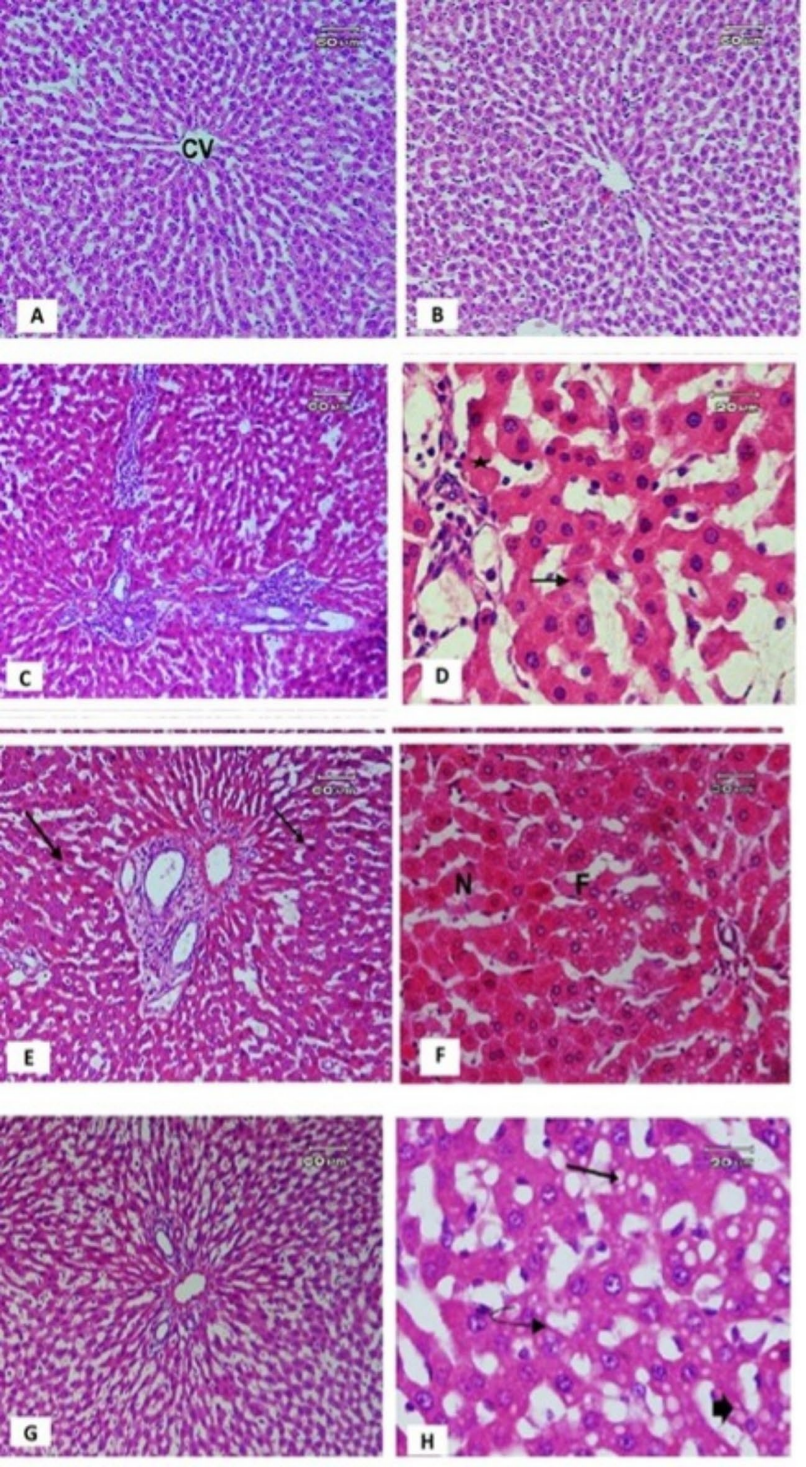
Section of rat liver (**A**) control showing normal structure of hepatic lobule, hepatic cells radiated from central vain (CV) and sinusoids in between, (**B**) treated with garden cress showing hepatic lobule almost normal, while some inflammatory cells infiltration in between sinusoids were noticed. (**C**) Methotrexate showing marked cellular infiltration and septa of fibrosis around portal tracts, shrinked hepatic cells with pyknotic small nuclei and eosinophilic cytoplasm. (**D**) High power of liver section treated with methotrexate showing apoptotic cells (arrow) and different form of nuclear damage represented in polymorphism (uneven size of the nuclei), pyknosis, karyolitic, karyorrhexis as well as necrotic areas (star)**.** (**E**) Methotrexate plus (200 mg/Kg) Garden cress extract showing some improvement represented in reduction in fibrosis around portal area, while the thickening of the portal tract with infiltration of inflammatory cells as well as dilation of sinusoid is present. (**F**) High power of liver section treated with methotrexate along with Garden cress at dose (200 mg/kg) showing hepatocyte vacuolization, fatty degeneration (F) and focal necrotic areas (N). (**G**): methotrexate plus Garden cress extract at dose (400 mg/Kg) showing much improvement manifested by reduction of connective tissue around portal tract, proliferation of bile ducts and cellular infiltration. Meanwhile the damage of hepatic cells and necrotic areas still present. (**H**): High power of section of liver treated with methotrexate plus Garden cress extract at dose (400 mg/Kg) showing of nuclear degeneration in the form of (pyknosis, karyolitic (curved arrow), as well as, marked vacuolar degeneration (arrow) and fatty degeneration (thick arrow) of hepatocytes still found (Hx and E).

The microscopic examination of the liver section of control rats (Fig. 7A) showed a normal structure of the hepatic lobule, with cords of hepatic cells radiating from the central vein separated by blood sinusoids. In contrast, the treated rats with Garden cress seed oil extraction showed hepatic lobules that were almost normal, except for some inflammatory cells infiltrating between the sinusoids (Fig. 7B). Animals treated with methotrexate (MTX) exhibited marked cellular infiltration and septa of fibrosis around portal tracts, with shrinking hepatic cells containing pyknotic small nuclei and eosinophilic cytoplasm (Fig. 7C). Various forms of nuclear damage were present, including apoptosis and polymorphism (uneven size of the nuclei), pyknosis, karyolysis, karyorrhexis, and necrotic areas (Fig. 7D).

The livers of animals treated with MTX and garden cress seed oil at a dose of 200 mg/kg showed some improvement, such as a reduction in fibrosis around the portal area, infiltration of inflammatory cells, and dilation of sinusoids (Fig. 7E). Hepatocyte vacuolization, fatty degeneration, and focal necrotic areas (Fig. 7F) were also present. In the group treated with MTX plus garden cress oil at a dose of 400 mg/kg, there was much improvement, with a reduction in connective tissue around the portal tract, proliferation of bile ducts, and cellular infiltration (Fig. 7G). However, nuclear degeneration in the form of pyknosis, karyolysis, as well as marked vacuolar degeneration and fatty degeneration of hepatocytes, were still observed (Fig. 7H).

**Fig. 8:**
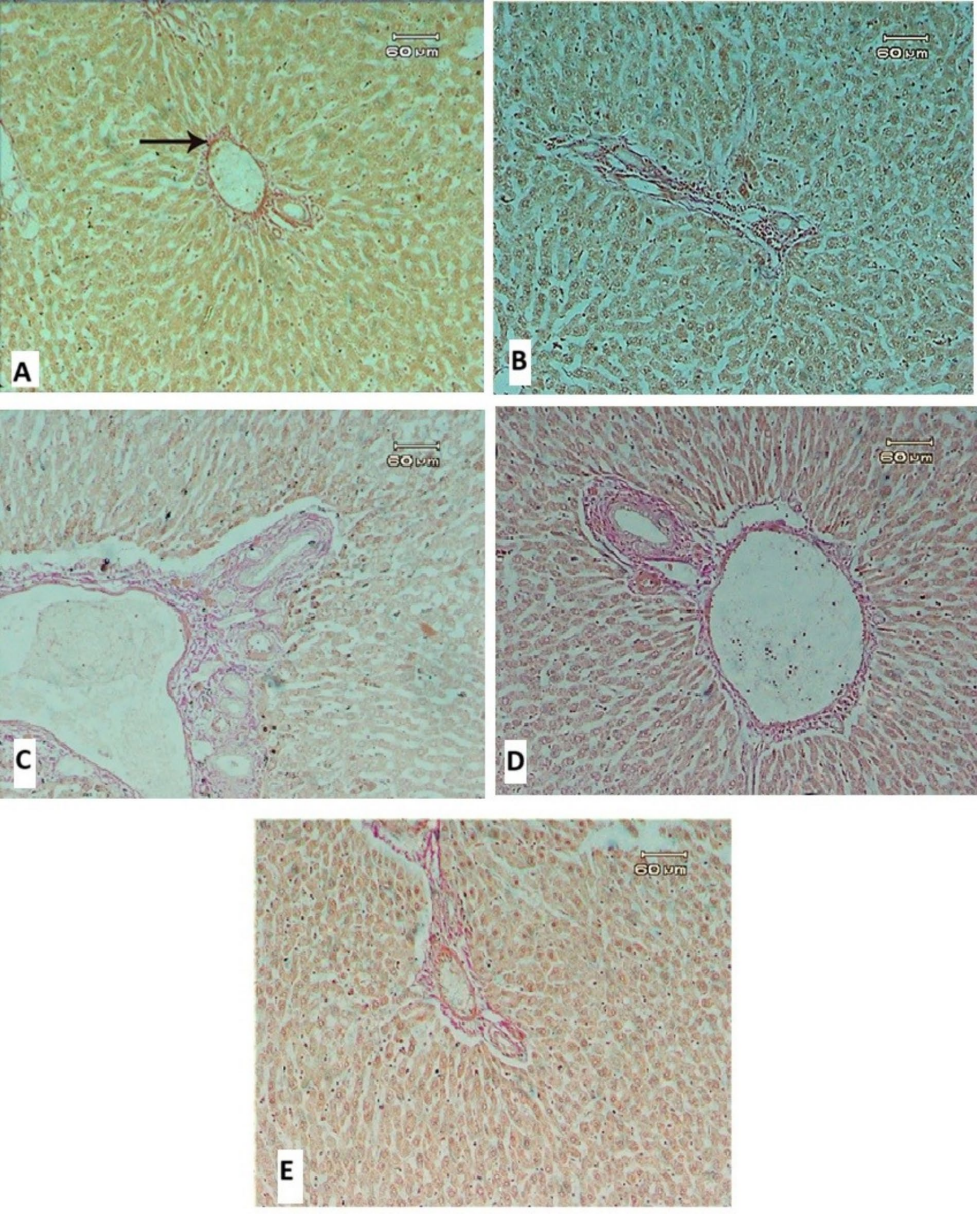
Image (**A**): Liver section of control rat showing normal distribution of fibrous tissue around portal area (arrow). (**B**) Liver section of rat treated with garden cress showing minimal amount of fibrous tissue and slights dilation in portal area. (**C**) Liver treated with methotrexate showing severe dilation of portal area and increase in fibrous tissues around portal tract, proliferation of bile ducts and massive cellular infiltration. (**D**) Liver treated with methotrexate pulse garden cress extraction at dose (200 mg/kg) showing some improvement represented in decrease in fibrous tissue while the dilation still present (**E**) Liver treated with methotrexate pulse Garden cress extraction at dose (400 mg/kg) showing much improvements represented in reduction in fibrous tissues around portal tract.

Van Gieson’s stain is a simple method used for differential staining of collagen in connective tissue. It gives collagen a pink color, similar to what is seen in fibrosis. In control animals, the liver showed a normal distribution of fibrous tissue around the portal area (Fig. 8A). However, livers treated with Garden cress exhibited a slight increase in fibrosis around the portal tract (Fig. 8B). The group treated with MTX showed a significant increase in fibrous tissues around the portal tract, along with bile duct proliferation, massive cellular infiltration, and severe dilation of the portal area (Fig. 8C). Animals treated with MTX in combination with garden cress extract at a dose of 200 mg/kg showed some improvement, with a decrease in fibrous tissue, although dilation was still present (Fig. 8D). The group treated with MTX and garden cress seed oil at a dose of 400 mg/kg showed even more improvement, with a reduction in connective tissue around the portal tract (Fig. 8E).

## Discussion

Methotrexate is the cornerstone of treatment for autoimmune diseases and a crucial part of managing inflammatory rheumatic disorders. Prescribers considering starting long-term methotrexate therapy for their patients have long been concerned about hepatotoxicity^27,28^. Lepidium sativum, or garden cress is an overlooked edible herb native to Egypt and commonly cultivated worldwide. The seed oil is reported to have beneficial health-promoting effects in metabolic disorders like diabetes and hyperlipidemia, as well as antioxidant, anti-inflammatory, and antirheumatic activities^29^. Other studies have reported in vitro and in vivo hepatoprotective effects, prevention of hepatocarcinogenesis, anticarcinogenic activity, and detoxification of carcinogens by the seed extracts/juice^30,31^. Garden cress (Lepidium sativum) seeds’ nutritional, ethnopharmacological, and medicinal relevance might be owed to their content of bioactive compounds and antioxidant properties^32^.

The phytochemical investigation of commercial GCO via GC/MS analysis revealed the presence of 67 unsaponifiable compounds [19 mono-/sesqui-/di-/and tri-terpenes (4.75%), 2 phenyl propanoids (0.5%), 3 tocopherols (0.08%), 2 lignans (2.47%), 6 sterols (1.59%), 35 alkane hydrocarbons (80.26%)]. These findings are consistent with previously reported literature^33–35^. The oil itself is odorless, but, its unsaponifiable fraction has a characteristic aromatic scent attributed to the detected mono- and sesqui-terpenes. Additionally, 6 fatty acids were identified, with oleic and linoleic acids being the major ones. Several metabolites (Carotane sesquiterpenes, carotol, daucol, bisabolene, cuparene and cupranene) were reported for the first time in this study.

According to the liver function analysis, MTX induced liver damage, as evidenced by elevated ALT, AST, and ALP enzyme activities. These findings were consistent with those of Abdul Kadhim et al.^36^, who linked the structural damage of the liver to increased levels of livers enzymes. Such enzymes are located in the cytoplasm and are released into the bloodstream following cellular damage, which signifies the onset of hepatotoxicity. In the investigation, we are studying the role of garden cress in attenuating the hepatotoxicity of MTX through inflammatory and apoptotic gene pathways as well as histopathological observations. Most drugs are metabolized by the liver and kidneys, making the liver susceptible to drug-induced liver injury (DILI)^37^. In hepatocytes, methotrexate (MTX) is converted to MTX-polyglutamate (MTX-PGs) by folylpolyglutamate synthase (FPGS) leading to apoptosis, fibrosis, oxidative stress, inflammation, and steatosis. The up-regulation of TNF-α observed in our study is a relevant biomarker associated with the inflammation pathway in MTX-induced hepatotoxicity. This significant increase in hepatic TNF-α may be due to MTX-PG-induced intracellular ROS, which in turn activate transcription factors like NF-kB and Nrf-2. Their nuclear translocation causes pro-inflammatory responses through the release of several inflammatory cytokines, such as TNF-α^2^. Consistent with our findings, previous studies have reported that the anti-inflammatory property of *L. sativum* was attributed to the lowering of TNF-α levels^38^. Ahmed et al.^39^ showed that Lepidium sativum polysaccharides (LSP) have the potential to modulate inflammatory mediator TNF-α in septic mice. Another study by Abdulmalek et al.^40^ showed that *L. sativum* seed extract could alleviate inflammatory and insulin sensitivity deviations in the liver of high-fat diet rats. Notably, our research findings demonstrated that GCO inhibited MTX-induced hepatic inflammation by lowering TNF-α mRNA expression. The anti-inflammatory effect of GCO is likely attributed to the antioxidant and/or anti-inflammatory activities of the reported bioactive components such as phenyl propanoids, α-Linolenic acid, sterols, and triterpenes^26^. Forms of vitamin E such as γ-tocotrienol, γ-tocopherol, and δ-tocopherol are potent natural therapeutic antioxidants with anti-inflammatory properties that help prevent many illnesses^41^. Both lignans (sesamin and sesamolin) are reported to have antioxidant, immunomodulatory and anti-inflammatory activities as well as beneficial health promoting effect on decreasing hepatic lipogenic activity through increasing fatty acid oxidation enzymes^42^. Furthermore, a recent study found that oleic and palmitic acids have anti-inflammatory and antimicrobial activities. They achieve this by reducing the expression of TNF in lipopolysaccharide-stimulated macrophages^43^.

Regarding apoptotic markers, MTX resulted in significantly higher expression levels of Bax, caspase-3, and P53 genes. Our results support previous studies that have shown increased expression levels of these genes in vivo in response to MTX^44–46^. During MTX-induced hepatotoxicity, an increase in oxidative stress leads to Bax translocation to the outer mitochondrial membrane, resulting in increased mitochondrial permeability and cytochrome c release into the cytosol. This activates downstream effector caspases, such as caspase-3^47^. Conversely, our results indicated that GCO downregulated the elevation of Bax, Caspase-3, and P53 induced by MTX in rat liver tissue, explaining its antiapoptotic action. These findings are consistent with Raish et al.^48^, who reported that *Lepidium sativum* ethanolic extract significantly down-regulated the expression levels of caspase-3 in a galactosamine/lipopolysaccharide-induced liver damage model. Additionally, oleic acid, a constituent of GCO, showed a decrease in the expression of caspase-3, Bcl-2, and Bax, thereby inhibiting hepatocyte apoptosis^48^.

The in silico docking study of the identified metabolites into TNF-α (pdb: 7JRA) and Caspase-3 (pdb: 3GJQ) target proteins suggested that the observed anti-inflammatory activity of GCO through inhibition of TNF-α enzyme could be attributed to tocopherols (α-/β-/ and γ), sesamolin lignan, and erucic acid. These compounds showed the highest binding affinity, with docking scores ranging from 9.52 to 10.73 kcal/mol, compared to the co-crystalized ligand (12.89 kcal/mol). Tocopherols, especially vitamin E, and sesamolin were reported to attenuate TNF-α gene expression and improve the treatment of hepatotoxicity^49^. Linoleic acid showed the highest docking score against caspase-3 protein (10.05 kcal/mol) compared to its co-crystalized ligand (11.01 kcal/mol), followed by other fatty acids. Kabakci and Bozkır et al.^45^ demonstrated that linoleic acid has a protective effect on methotrexate-induced liver toxicity by modulating the expression of apoptotic pathway mediators such as Bax, Bcl-2 and Caspase-3. Linoleic acid was found to attenuate acute liver injury associated with lipopolysaccharide by alleviating histopathological abnormalities and liver enzymes, as well as inhibiting proinflammatory markers such as TNF-α and IL-6^50^.

The histological analyses revealed significant liver damage in the MTX-treated group, including interface necrosis, apoptotic cells, and central zone lymphocyte infiltration. These alterations are corroborated by previous research^51^. Morsy et al.^13^ demonstrated that rats receiving MTX showed high collagen deposition in their liver tissue, primarily leading to hepatic fibrosis. Taskin et al.^52^ confirmed that MTX exhibited many pathological anomalies in the liver, including hepatocyte necrosis, fibrosis, and an increase in cellular infiltration. In response to apoptosis, cells undergoing necrosis lose membrane integrity and release their intracellular components, serving as danger signals that trigger inflammation^53^. Van Loo and Bertrand^54^ discovered that TNF induces inflammatory responses by directly stimulating the expression of inflammatory genes and indirectly by promoting inflammatory immune responses and cell death. These findings support the upregulation of TNF-α mRNA expression in groups treated with MTX.

Meanwhile, the present study indicated that garden cress restores the histopathological alterations induced by MTX. According to Zamzami et al.^55^, *Lepidium sativum* enhanced liver function in CCl4-treated New Zealand white rabbits by reversing the liver histopathologic alterations. Additionally, Ibrahim et al.^56^ reported that a daily dose of 400 mg/kg b.w. of garden cress ethanolic extract has hepatoprotective, antioxidant, and anti-steatosis properties in rats. Garden cress has been found to have potential benefits for liver tissue regeneration, where bioactive compounds in the seeds are thought to promote the growth of new liver cells, aiding in the restoration of injured liver tissue^57^. On the other hand, the liver of rats treated with garden cress oil showed very few inflammatory cells penetrating between the sinusoids. This finding was supported by Abuelgasim et al., who reported that the rats administered 400 mg/kg (Bwt) showed scarred vacuolated cells and hepatic congestion^58^.

## Conclusion

All of our data points to the fact that GCO protects the liver from MTX-induced damage. This is partially explained by its strong anti-inflammatory, anti-fibrotic, and anti-apoptotic properties. These properties are probably attributed to the active compounds present in GCO that were recognized from our in-silico study as compounds for attenuating the inflammatory and apoptosis reactions in the liver by inhibiting TNF-α and caspase-3, as proven by gene expression results.

## Materials and methods

### Chemicals

Methotrexate was obtained from EIMC United Pharmaceuticals Company (Cairo-Egypt). Cold-pressed Garden cress oil was purchased from Haraz Co., Cairo, Egypt. All other chemicals were of analytical grade and acquired from standard marketable suppliers.

GC/MS analysis of lipid content in garden cress seed oil

Saponification of garden cress oil (GCO) (8 g) was performed by refluxing for 6–8 h with alcoholic KOH, followed by extraction of unsaponifiable matter with ether, yielding 0.7 g upon evaporation to dryness. Fatty acids were methylated through reflux with a 2 M HCl solution in methanol for 3–4 h, then extracted with ether and evaporated to dryness, yielding 4.8 gm as fatty acid methyl esters (FAME)^59^.

Both fractions were then analyzed via a Shimadzu GCMS-QP2010 (Kyoto, Japan) equipped with an Rtx-5MS fused bonded column (30 m × 0.25 mm i.d. × 0.25 μm film thickness) (Restek, USA) with a split–spitless injector. The following guidelines were established: the initial temperature of the column was maintained at 50 °C for three minutes (isothermal), then it was increased to 300 °C at a rate of 5 °C per minute and held there for ten minutes (isothermal). Even though the injector temperature was established at 280 °C. The flow rate of the helium carrier gas was 1.37 ml/min. The following parameters were applied to all mass spectra recordings: Ion source temperature: 220 °C; ionization voltage: 70 eV; filament emission current: 60 mA. Split mode injections were used with diluted samples (1% v/v; split ratio: 1:15). Identification of compounds was achieved by comparing their retention index (RI) and mass spectral data with NIST/Wiley, Pherobase, and other literature sources^60^.

### Molecular docking study of identified compounds

The 3D coordinates of the detected phytoconstituents were obtained in SDF format from the PubChem database. After being energy-minimized to a gradient of 0.01 kcal/mol Å in the gas phase using the MMFF94x Force Field, the data were saved in PDBQT format. Human TNF-α (PDB ID: 7JRA) and human caspase-3 (PDB ID: 3GJQ) co-crystal structures were obtained from the Protein Data Bank (https://www.rcsb.org). Using MGL Tools v1.5.7, all target receptors were prepared by deleting water molecules and other hetatoms, adding polar hydrogens, and assigning Kollman charges. The receptors were then saved in PDBQT format. Grid boxes measuring 25 × 25 × 25 Å were positioned at the co-crystallized ligands to encompass the entire binding sites of the target receptors. To perform all docking computations, AutoDock Vina, an open-source program, was utilized. The docking poses were ranked according to their docking scores, and the pose with the best energy was selected. Using Discovery Studio Visualizer v21.1.0.20298, the interactions between the selected compounds and the target proteins were examined^61^.

### In vivo experimental design

Thirty adult male Sprague–Dawley albino rats, weighing between 100 to 150 g, were obtained from the animal facility at the National Research Center in Giza, Egypt. The rats were housed in a room with controlled environmental conditions, including a 12-h light/12-h dark cycle and a temperature of 22 °C. They were kept in clear plastic cages with stainless steel wire tops, and provided with rat feed pellets and unrestricted access to water. The study was approved by the Ethics Committee at the National Research Center (Approval No. 09410125). The research methods were carried out following relevant guidelines and ARRIVE guidelines.

The rats were randomly divided into five groups, each consisting of six animals. The animals were given a week to acclimate before the start of the study. The groups were as follows: Group I: rats received oral gavages of saline as a negative control. Group II: animals were orally administered Garden cress oil (400 mg/kg/day) for 28 days as a vehicle group. Group III: animals were intraperitoneally injected with methotrexate at a dose of 5 mg/kg/day for 7 days following the protocol of Demiryilmaz et al.^62^. Groups IV & V: animals were injected with methotrexate following the same protocol as Group III. On the eighth day, they were orally administered garden cress oil at doses of 200 and 400 mg/kg once daily for 28 days, as described by Yogesh et al.^63^.

### Blood and tissue sampling

At the end of the experiment, rats were anesthetized with an intraperitoneal injection of xylazine (5 mg/kg b.w.) and ketamine (50 mg/kg b.w.) (Sigma Aldrich, USA; Cat. No. K113). Following this, blood samples were taken from the heart cavity in heparinized glass tubes and centrifuged for ten minutes at 5000 rpm. Aliquots of the plasma were kept at -80 °C until analysis, once the livers had been removed and rinsed with cold saline. After that, portions of the liver were frozen in liquid nitrogen and kept at -80 °C for the real-time quantitative PCR (RT-qPCR) study. A portion of the tissue samples was kept in 10% formalin buffer for histopathological analysis.

### Liver enzymes assessment

Serum alanine aminotransferase (ALT), aspartate aminotransferase (AST), and alkaline phosphatase (ALP) activities were measured in all blood samples. These enzymes were tested using colorimetric commercial kits from Bio Diagnostic (Egypt) according to the manufacturer’s instructions.

### RNA isolation, cDNA synthesis, and reverse transcription polymerase chain reaction (RT-PCR) analysis

Isolated liver samples were homogenized in an Easy Red Total RNA Extraction Kit (Intronbio, Korea), and RNA was extracted following the manufacturer’s instructions. The yield and quality of isolated RNAs were assessed through gel electrophoresis and spectrophotometric measurement. The RNA was then treated with the RNase-free DNase kit (Thermo Scientific) and cDNA was synthesized via reverse-transcription as per the manufacturer’s instructions (Thermo Scientific, China). Gyceraldehyde-3-phosphate dehydrogenase (GADPH) was used as the internal control and the expression of four genes (Bcl-associated X protein (Bax), cysteine aspartic acid specific protease 3 (Caspase-3), tumor necrosis factor alpha (TNF-α) and tumor-suppressor protein (P53) was evaluated in the study. RT-qPCR was conducted using the Stratagene Mx3000P Real-Time PCR System (Agilent Technologies, USA) and carried out in a 25 µL reaction containing cDNA, TOPreal™qPCR 2X PreMIX (SYBR Green with low ROX) (Enzynomics), forward and reverse primers (10 pmol/μl) (Macrogen), and free water nuclease. The gene expression levels were calculated using the 2-ΔΔCt method^64^. The primers used for RT-qPCR above are listed in Table 4.

**Table 4:** Primers of candidate genes.

Gene	Primer forward	Primer reverse	Product size	Accession No
Caspase	ATTGACACAATACACGGGATCTGT	AAATTCAAGGGACGGGTCAT	183	NM_012922.2
Bax	AGA GGA TGA TTG CTG ATG TGG	CCC AGT TGA AGT TGC CGT	93	NM_017059.2
P53	GCA GAG TTG TTA GAA GGC	TTG AGA AGG GAC GGA AGA	138	NM_030989.4
TNF	CCACCACGCTCTTCTGTCTAC	ACCACCAGTTGGTTGTCTTTG	256	NM_012675.3
GAPDH	AACTTTGGCATTGTGGAAGG	ACACATTGGGGGTAGGAACA	223	NM_017008.4

### Histological methodology

#### Hematoxylin and eosin stain

The liver specimens were collected, fixed in a 10% buffered formalin (Thermo Fisher Scientific, Waltham, MA) at room temperature for one to three days, and embedded in paraffin. Subsequently, 5 μm thick paraffin tissue sections were subjected to standard procedures, including deparaffining, hematoxylin and eosin staining, dehydration, and mounting were prepared and stained with H&E stain following the method of Drury and Wallington^65^. Using an optical microscope (Olympus, IX53, Tokyo, Japan), the stained slides were inspected and captured on camera.

#### Van Gieson stain

Van Gieson stain was used to evaluate liver fibrosis, following the protocol outlined by Chen et al.^66^. Briefly, paraffin-embedded liver sections were deparaffinized and hydrated in distilled water. They were then stained with Wright’s Working Hematoxylin for 10 min and washed in distilled water. The slides were further stained with Van Gieson solution for 3 min, followed by gradient dehydration in 95% alcohol, absolute alcohol, and 2 changes in xylene before mounting with DPX for investigation.

### Statistical analysis

The data obtained were presented as means ± standard error of the means (SEM) (*n* = 3) and analysis was performed using the SPSS 16.0 program (SPSS Inc., Chicago, IL, USA). One way analysis of variance method was used to evaluate the statistical differences. Differences among groups were considered statistically significant at *p* values ≤ 0.05.

## Data Availability

Data is provided within the manuscript file.
